# Rubrivirga marina gen. nov., sp. nov., a member of the family *Rhodothermaceae* isolated from deep seawater

**DOI:** 10.1099/ijs.0.055467-0

**Published:** 2013-08

**Authors:** Sanghwa Park, Jaeho Song, Susumu Yoshizawa, Ahyoung Choi, Jang-Cheon Cho, Kazuhiro Kogure

In the protologue of this paper (doi:10.1099/ijs.0.046318-0), the type strain SAORIC-28^T^ is erroneously cited as SAROIC-28^T^.

doi:10.1099/ijs.0.055467-0

